# Magnetoreceptive CRY/MagR complexes: linking circadian redox signalling to protein aggregation in Alzheimer’s and Parkinson’s disease

**DOI:** 10.5599/admet.3366

**Published:** 2026-05-28

**Authors:** Mozhgan Alipour, Behnam Hajipour-Verdom, Faria Ashrafi, Sara Rahmati Roodsari, Shabnam Nohesara, Alireza Zali, Farzad Ashrafi

**Affiliations:** 1 Functional Neurosurgery Research Center, Research Institute of Functional Neurosurgery, Shohada Tajrish Comprehensive Neurosurgical Center of Excellence, Shahid Beheshti University of Medical Sciences, Tehran, Iran; 2 Department of Integrative Oncology, Breast Cancer Research Center, Motamed Cancer Institute, Academic Center for Education, Culture and Research (ACECR), Tehran, 1517964311, Iran; 3 Department of Biology, University of Massachusetts Amherst, Amherst, MA 01003, USA; 4 Department of Medicine (Biomedical Genetics), Boston University Chobanian and Avedisian School of Medicine, Boston, MA 02118, USA

**Keywords:** Cryptochrome, magnetoreceptor, oxidative stress, circadian rhythms

## Abstract

**Background and purpose:**

Neurodegenerative disorders such as Alzheimer’s disease (AD) and Parkinson’s disease (PD) pose an escalating challenge to neuroscience, as disease-modifying therapies remain elusive despite substantial advances in molecular and cellular understanding. These disorders share convergent pathological features, including protein misfolding and aggregation, mitochondrial dysfunction, oxidative stress, impaired proteostasis, and disruption of circadian regulation. Identifying integrative frameworks that connect these processes is therefore essential for advancing conceptual models of neurodegeneration. This review examines magneto-proteins, with a particular focus on CRY/MagR-based magnetoreceptor complexes, as emerging biological systems that may intersect with key molecular pathways implicated in AD and PD.

**Experimental approach:**

We synthesized literature from neuroscience, biophysics, and circadian biology to evaluate the potential relevance of magnetoreceptor mechanisms to AD and PD pathology. We first summarized the core neuropathological mechanisms underlying both diseases, including amyloid-β and tau pathology in AD and α-synuclein aggregation and dopaminergic vulnerability in PD. We then outlined the biophysical foundations of magneto-protein function, emphasizing cryptochrome-mediated radical pair mechanisms, iron-sulphur cluster-dependent magnetic sensitivity, and their established roles in redox signalling and circadian biology.

**Key results:**

Accumulating experimental evidence from cellular and animal models suggests that CRY/MagR-associated pathways can modulate oxidative stress, mitochondrial bioenergetics, protein aggregation dynamics, autophagic processes, and circadian control of neuronal metabolism. These processes closely overlap with molecular determinants of neuronal vulnerability in AD and PD. However, direct validation in mammalian and human systems remains limited and controversial, representing a critical knowledge gap.

**Conclusion:**

The mechanistic convergence between magnetoreceptor biology and neurodegenerative pathology warrants critical evaluation but remains largely speculative in humans. By integrating findings across disciplines, this review positions CRY/MagR-based magneto-proteins as a conceptual platform for exploring how magnetic field-responsive molecular systems may inform our understanding of neurodegenerative disease mechanisms, while emphasizing the need for rigorous mammalian validation.

## Introduction

Neurodegenerative diseases (NDs) such as Alzheimer’s disease (AD) and Parkinson’s disease (PD) represent a growing global health challenge, largely driven by an aging population. AD is the most common cause of dementia, accounting for approximately 60 to 70 % of cases, and currently affects more than 55 million people worldwide, a number expected to triple by 2050. PD is the second most prevalent NDs, with over 8.5 million individuals affected globally, and its incidence has more than doubled in the last 25 years, making it the fastest-growing neurological disorder. Together, these diseases contribute to substantial disability, dependence, and mortality in older adults. This neuronal degeneration disrupts communication within the nervous system, impairing cognitive, motor, and/or autonomic functions depending on the affected brain regions [1,2].

The socio-economic burden of AD and PD is equally profound. The global cost of dementia alone was estimated at over US$1.3 trillion in 2019 and is projected to surpass US$2.8 trillion by 2030, reflecting not only direct medical expenses but also the hidden costs of informal care provided by families. Similarly, PD imposes considerable strain due to long-term disability, loss of productivity, and the need for continuous clinical management. Beyond financial costs, these disorders diminish quality of life for both patients and caregivers, often resulting in emotional, physical, and psychological stress that extends far beyond the affected individuals themselves [3,4].

Despite their impact, current therapeutic options for AD and PD remain limited. Available treatments primarily offer symptomatic relief, such as cholinesterase inhibitors and memantine for AD or dopaminergic therapies for PD, which temporarily improve cognitive or motor function. However, these interventions do not halt or reverse the underlying neurodegenerative processes. Numerous clinical trials targeting disease-modifying pathways, such as amyloid clearance in AD or neuroprotective strategies in PD, have thus far yielded limited success [5,6]. This therapeutic gap underscores the urgent need for innovative approaches that can address the molecular underpinnings of protein misfolding, aggregation, and neuronal dysfunction characteristic of these disorders.

In this context, the emergence of magneto-proteins represents a novel frontier in the search for next-generation therapies for neurodegenerative diseases. Magneto-proteins are engineered or naturally occurring proteins that can respond to external magnetic fields, enabling remote, non-invasive, and targeted modulation of neuronal activity. Unlike conventional pharmacological agents that rely on systemic delivery and often lack cellular specificity, magneto-proteins allow spatiotemporal control over neuronal signalling, opening new opportunities for precise therapeutic interventions in disorders such as AD and PD [7,8].

This concept lies at the intersection of bioengineering, neuroscience, and nanomedicine. By integrating magnetic field-responsive elements with neuronal proteins, researchers are developing innovative tools that not only deepen our understanding of brain function but also hold translational potential as disease-modifying strategies. The rise of magneto-proteins thus represents a promising step toward bridging molecular mechanisms with clinical applications, offering a new paradigm for non-invasive neuromodulation in complex brain disorders.

## Alzheimer’s disease

AD is the most common form of dementia. This non-communicable disorder begins in the hippocampal region of the brain and gradually spreads to other areas over time. Its progression manifests as impaired thinking ability, motor skills, learning, memory, and language functions, all of which arise from neuronal death and ultimately lead to severe dementia in multiple brain regions [9,10].

AD is characterized by two major pathological hallmarks: the formation of amyloid plaques and neurofibrillary tangles (NFTs), both of which contribute to neuronal degeneration. Amyloid plaques, also referred to as senile plaques, are formed by the amyloid-β (Aβ) peptide when it aggregates into dense fibrils outside neurons. This peptide is a byproduct of the amyloid precursor protein (APP), which plays an essential role in neuronal development. APP and the amyloid precursor-like proteins APLP1 and APLP2 in mammals, along with APPL in Drosophila, constitute a protein family whose members each span the membrane once and possess a large extracellular domain. Within this family, only APP can generate the amyloidogenic fragment [11-13].

Eight isoforms of APP have been identified, of which three are the most common: the 695-amino-acid, 751-amino-acid, and 770-amino-acid variants. The 695-amino acid isoform is predominantly expressed in the central nervous system, whereas the 751 and 770-amino acid isoforms are expressed more broadly across tissues. A major portion of the Aβ sequence is located within the extracellular N-terminal region of APP, while a smaller segment resides within its transmembrane domain. APP also contains an intracellular C-terminal region that includes a highly conserved motif composed of the amino acids Tyr, Thr, Pro, Asn, Glu, and Tyr, referred to as the “YENPTY” sequence [14,15].

APP undergoes proteolytic processing via two alternative and mutually exclusive pathways. In the non-amyloidogenic pathway, α-secretase cleaves APP within the Aβ region, releasing a soluble extracellular fragment (sAPPα) and generating an 83-amino-acid C-terminal fragment (C83). Subsequent cleavage of C83 by γ-secretase produces the non-pathogenic P3 peptide along with the APP intracellular domain (AICD). In contrast, the amyloidogenic pathway is initiated by β-secretase cleavage, yielding a soluble extracellular fragment (sAPPβ) and a 99-amino-acid C-terminal fragment (C99). γ-Secretase processing of C99 then results in the generation of Aβ peptides of varying lengths, together with the same intracellular fragment, AICD, thereby linking this pathway to AD pathology [14,16,17].

APP plays a crucial role in a wide range of physiological processes, including cell-cell adhesion and interactions, binding to the extracellular matrix and cytoskeleton, regulation of cell growth and survival, neuronal migration and motility, synapse formation, neurite outgrowth, synaptic plasticity, and the proper distribution of neurons within the brain [18]. In addition to these essential functions, APP undergoes proteolytic processing mediated by secretase enzymes, a process that can lead to the generation of Aβ peptides under amyloidogenic conditions [19]. Within the full-length APP molecule, the Aβ region adopts an α-helical conformation; however, following proteolytic cleavage and peptide release, this region undergoes a conformational transition into β-sheet-rich structures that favour aggregation [20]. Aβ peptides range from 36 to 43 amino acids in length, with Aβ40 and Aβ42 being the most abundant and biologically relevant species, the latter being particularly associated with increased aggregation propensity and neurotoxicity [21].

Aβ peptides are inherently hydrophobic, a property that strongly favours their tendency to self-associate through hydrophobic interactions, leading to progressive assembly into a spectrum of aggregated species. Initially, monomeric Aβ molecules misfold and oligomerize into low-molecular-weight oligomers, which can further associate into protofibrils and eventually into highly ordered fibrils that deposit extracellularly as amyloid plaques in AD brain tissue. This hierarchical aggregation process is driven by β-sheet-rich conformations stabilized by inter- and intramolecular interactions, with hydrophobic segments of the peptide playing a critical role in nucleation and growth of these assemblies. Importantly, soluble oligomers and protofibrillar assemblies, rather than the insoluble plaque cores themselves, are now widely recognized as the principal neurotoxic species, capable of interacting with neuronal membranes and receptors, disrupting synaptic function and plasticity, and initiating downstream pathways that contribute to synaptic degeneration and cognitive decline in AD [22-24].

A β-strand is a structural element of a polypeptide chain comprising approximately 3 to 10 amino acids arranged in an extended conformation. Multiple β-strands can associate laterally via inter-strand hydrogen bonds to form β-sheets, which are stabilized by regular hydrogen-bonding patterns between backbone amide and carbonyl groups. In the context of amyloidogenic proteins, the supramolecular assembly of β-sheets into stacked arrays is a fundamental step in the formation of amyloid fibrils and higher-order protein aggregates observed in amyloidoses, including AD. In these fibrils, monomeric Aβ peptides align perpendicular to the fibril axis, adopting a characteristic cross-β architecture in which β-strands from adjacent molecules run parallel to one another, and their lateral associations are reinforced by extensive hydrogen bonding networks. Although Aβ peptides may accumulate intracellularly under certain pathological conditions, their predominant localization in AD is extracellular, where they contribute to plaque deposits and disrupt normal tissue architecture [25,26].

The Aβ peptide can undergo several post-translational modifications (PTMs), including oxidation, racemization, isomerization, pyroglutamate formation at glutamate residues, and phosphorylation. Phosphorylated Aβ at Ser8 and pyroglutamate-modified Aβ exhibit similar aggregation behaviour and strongly promote the formation of oligomers and fibrillar Aβ assemblies. Such modifications increase the biochemical stability of Aβ and elevate its concentration within the brain [27].

Between monomeric Aβ peptides and mature fibrillar structures, a range of intermediate assemblies exists that either arise during fibril formation or represent structural transition states. In the Alzheimer’s brain, these intermediates include protofibrils and low‑ and high‑molecular-weight oligomers. Dimers participate in the fibril-forming pathway, whereas trimers and globulomers remain stable off‑pathway species that do not proceed toward fibril formation [28].

The role of soluble oligomeric species of Aβ in fibril formation was proposed more than two decades ago, and their toxic effects are well established today. In the Alzheimer’s brain, oligomers accumulate within astroglia and are subsequently taken up by microglia. Intraneuronal accumulation of Aβ is less frequently observed in elderly individuals with Down syndrome and in the late stages of AD, where amyloid plaques are predominantly found extracellularly. In contrast, during the early stages of the disease and in individuals with Down syndrome, intraneuronal accumulation of Aβ oligomers has been reported [29,30].

Extracellular aggregates of Aβ peptides assemble into senile plaques, which are a pathological hallmark observed throughout multiple regions of the AD brain. This aberrant deposition reflects an imbalance between Aβ production and elimination, rather than solely increased synthesis. In early AD, cerebrospinal fluid concentrations of Aβ-particularly Aβ42-are reduced, while insoluble Aβ42 accumulates within brain parenchyma, indicating impaired clearance mechanisms [31,32].

Several enzymatic and transporter-mediated systems contribute to extracellular Aβ removal. Neprilysin (NEP) and insulin-degrading enzyme (IDE) are key Aβ-degrading proteases that metabolize soluble and aggregated Aβ peptides in the extracellular space, and reductions in their activity correlate with increased plaque burden in AD. Additionally, efflux transporters at the blood-brain barrier, including the ATP-binding cassette transporter P-glycoprotein (ABCB1) and the low-density lipoprotein receptor-related protein 1 (LRP1) facilitate transcytosis of Aβ from brain interstitial fluid into the circulation. In contrast, the receptor for advanced glycation end products (RAGE) mediates the influx of circulating Aβ into the brain. Alterations in the expression or function of these clearance components, namely decreased NEP/IDE activity, downregulation of P-glycoprotein and LRP1, and upregulation of RAGE impair Aβ efflux, promoting extracellular accumulation and plaque formation characteristic of AD pathology [33-35].

Abnormal, extracellular, fibrillar protein deposits found in tissues and organs are collectively referred to as amyloid. Amyloid is insoluble, adopts a β-sheet-rich fibrillar structure, and typically lacks structural, supportive, or motile functions, but it is associated with the pathology observed in a wide range of disorders collectively known as amyloidoses. These diseases, including AD, spongiform encephalopathies, and type II diabetes, are progressive conditions with high morbidity and mortality [36,37].

Aβ plays several physiological roles in the body, including enhancing synaptic plasticity, learning, and memory by promoting long-term potentiation in the hippocampus; modulating calcium dynamics; exerting antimicrobial and antiviral activity; facilitating recovery after brain injury; sealing blood-brain barrier leaks; and potentially suppressing cancer through effects on tumour growth and apoptosis. Additional proposed functions include regulation of cholesterol transport, kinase activation, transcriptional modulation, and participation in apoptosis. An imbalance between Aβ production and its clearance from brain tissue leads to extracellular accumulation of Aβ. Aβ also reduces ATP production and disrupts mitochondrial function, and this mitochondrial impairment contributes to the progression of AD [38,39].

NFTs are one of the principal neuropathological hallmarks of AD and play a critical role in neuronal dysfunction and death. These tangles consist of intracellular aggregates of abnormally hyperphosphorylated tau protein that assemble into paired helical filaments and straight filaments within neurons. Under normal physiological conditions, tau stabilizes microtubules and supports axonal transport; however, excessive phosphorylation reduces its affinity for microtubules, leading to cytoskeletal destabilization and impaired neuronal function. In AD brains, tau is approximately three to four times more hyperphosphorylated than in healthy brains and aggregates into the filamentous structures that form NFTs [40,41].

To date, more than 70 phosphorylation sites have been identified on tau, but the most critical residues involved in abnormal phosphorylation include Thr18, Ser199, Ser202, Thr205, Thr231, Ser396, and Ser422. The accumulation of hyperphosphorylated tau disrupts microtubule stability, impairs axonal transport, induces oxidative stress and mitochondrial dysfunction, and ultimately contributes to synaptic failure and neuronal degeneration. Among the various kinases implicated in tau dysregulation, glycogen synthase kinase-3 (GSK3), particularly its β isoform (GSK3β), is considered the principal enzyme mediating tau phosphorylation and hyperphosphorylation [42,43].

## Parkinson’s disease

PD is a progressive neurodegenerative disorder and is widely recognized as the second most common neurodegenerative disease after AD. PD affects millions of individuals globally and poses a significant burden on patients, caregivers, and healthcare systems due to its chronic and progressive nature. The pathological hallmark of PD is the progressive degeneration of dopaminergic neurons in the substantia nigra pars compacta (SNpc), resulting in a marked reduction in striatal dopamine levels and impaired basal ganglia circuitry. Dopamine deficiency underlies the characteristic motor dysfunctions of the disease and contributes to numerous non‑motor manifestations [44].

Clinically, PD is characterized by cardinal motor symptoms including bradykinesia (slowness of movement), resting tremor, rigidity, and postural instability, which progressively worsen with disease advancement. In addition to motor dysfunction, PD patients frequently experience a spectrum of non‑motor symptoms, such as cognitive impairment, mood disorders (*e.g.* depression, anxiety), autonomic dysfunction (*e.g.* orthostatic hypotension), sleep disturbances (*e.g.* REM sleep behaviour disorder), and olfactory deficits, often appearing in prodromal or early stages of the disease. These non‑motor features substantially impact quality of life and may precede motor signs by years [45].

PD is neuropathologically defined by two principal hallmarks: the selective loss of dopaminergic neurons in the SNpc and the presence of Lewy bodies (LBs). LBs are neuronal inclusions that develop intracellularly and are primarily composed of misfolded and aggregated α‑synuclein, reflecting a core pathological feature of PD and related synucleinopathies [46].

α‑synuclein is a 140-amino acid presynaptic protein encoded by the SNCA gene. Under physiological conditions, α‑synuclein exists predominantly as a soluble, intrinsically disordered monomer in the cytosol, but it can adopt α‑helical conformations upon association with synaptic vesicles and lipid membranes. In this state, it plays important roles in synaptic vesicle dynamics, including modulation of vesicle mobility, clustering, and the assembly of presynaptic machinery essential for efficient neurotransmitter release and synaptic homeostasis [46-48].

In healthy neurons, α‑synuclein contributes to presynaptic regulation by interacting with vesicle‑associated proteins such as VAMP2 and components of the SNARE complex, facilitating vesicle docking and neurotransmitter exocytosis. Its conformational flexibility enables dynamic engagement with membranes and protein partners critical for maintaining synaptic function and dopaminergic signalling [47].

In humans, α-synuclein is a member of the synuclein protein family, which also includes β-synuclein and γ-synuclein. Although β- and γ-synucleins share considerable structural similarity with α-synuclein, only α-synuclein exhibits a strong propensity for pathological aggregation, a defining feature of PD and related synucleinopathies. The aggregation behaviour and cytotoxicity of α-synuclein are critically regulated by PTMs, including phosphorylation, ubiquitination, nitration, sumoylation, and C-terminal truncation. Among these, phosphorylation at Ser129 is the most abundant modification detected in LBs and is strongly associated with pathological aggregation and neurotoxicity. In contrast, tyrosine phosphorylation at Tyr125, Tyr133, and Tyr136 has been reported to modulate α-synuclein aggregation dynamics and may contribute to neuroprotective signalling pathways [49,50].

α-synuclein undergoes a stepwise misfolding and aggregation process, progressing from monomeric forms to higher-order assemblies. Under pathological conditions, monomeric α-synuclein self-assembles into soluble oligomers, which can range from small dimers and trimers to larger multimeric species, before elongating into protofibrils and eventually forming insoluble mature fibrils that constitute the core of LBs. Soluble oligomeric intermediates are widely considered the most neurotoxic species in the aggregation continuum, as they disrupt multiple cellular processes. These toxic oligomers interfere with synaptic vesicle trafficking, impair dopamine release, disrupt calcium homeostasis, and induce oxidative stress, all of which contribute to mitochondrial dysfunction and neuronal injury, thus playing a critical role in PD pathogenesis [51,52].

Structurally, α-synuclein is composed of three distinct domains: The N-terminal amphipathic region (residues 1-60), which mediates membrane binding and α-helix formation; the non- Aβ component (NAC) domain (residues 61-95), which is hydrophobic and crucial for β-sheet stacking and aggregation into fibrils; and the C-terminal acidic region (residues 96-140), which regulates chaperone interactions, PTMs, and aggregation propensity. The NAC domain drives fibrillization due to its hydrophobic character, whereas the acidic C-terminal region modulates α-synuclein’s interactions with metal ions, kinases, phosphatases, and molecular chaperones, thereby influencing both physiological function and pathological aggregation [53,54].

Mitochondrial dysfunction is a central feature of PD pathogenesis. Impairment of mitochondrial complex I activity, particularly in the substantia SNpc, leads to reduced ATP production, increased generation of reactive oxygen species (ROS), and oxidative damage to lipids, proteins, and DNA. These mitochondrial deficits promote α-synuclein aggregation and exacerbate dopaminergic neuronal death. Additionally, α-synuclein interacts with mitochondria-associated membranes, modulating calcium buffering, mitochondrial dynamics (fission and fusion), and mitophagy. Mutations in PINK1 (PTEN-induced kinase 1) and Parkin impair mitophagy, resulting in the accumulation of dysfunctional mitochondria and increased vulnerability to oxidative stress. Similarly, DJ-1 mutations compromise antioxidant defences, further heightening neuronal susceptibility to degeneration [55,56].

Multiple kinase pathways regulate α-synuclein phosphorylation and influence PD progression. Key kinases, including GSK3β, casein kinase 1 and 2 (CK1/CK2), polo-like kinase 2 (PLK2), and leucine-rich repeat kinase 2 (LRRK2), have been identified as major modulators of Ser129 phosphorylation. Hyperactivation of these kinases is associated with enhanced α-synuclein aggregation, impaired synaptic function, and increased neuronal death. In contrast, phosphatases such as PP2A and PP2B counteract phosphorylation, underscoring the importance of dynamic kinase-phosphatase regulation in maintaining neuronal homeostasis and modulating PD pathology [57].

The ubiquitin-proteasome system and the autophagy-lysosomal pathway are critical for α-synuclein clearance. Impairment of proteasomal degradation or lysosomal dysfunction results in cytoplasmic accumulation of α-synuclein, formation of oligomers and fibrils, and subsequent neuronal toxicity. Chaperone-mediated autophagy selectively degrades monomeric α-synuclein via LAMP2A, while macroautophagy facilitates the removal of aggregated species. Mutations in GBA1, which encodes glucocerebrosidase, compromise lysosomal function and exacerbate α-synuclein accumulation, linking lysosomal dysfunction directly to PD pathogenesis [58,59].

LBs, the pathological hallmark of PD, follow Braak staging in their distribution. α-Synuclein accumulation initially occurs in the olfactory bulb and lower brainstem, corresponding to prodromal symptoms such as hyposmia and constipation. This is followed by involvement of the midbrain, leading to motor symptoms including bradykinesia and rigidity, and eventually spreads to cortical regions, contributing to cognitive decline and dementia. Structurally, LBs are composed of a dense core of fibrillar α-synuclein, surrounded by membranous organelles, vesicles, and ubiquitinated proteins, reflecting impaired protein homeostasis and cellular stress responses [60].

α-Synuclein also performs essential physiological functions, including regulation of synaptic vesicle pools, dopamine synthesis, vesicle recycling, synaptic plasticity, and protection against oxidative stress. Disruption of these functions-whether due to misfolding, PTMs, or impaired clearance-contributes to the neurodegenerative cascade in Parkinson’s disease. The interplay between mitochondrial dysfunction, oxidative stress, α-synuclein aggregation, kinase signalling, and impaired protein degradation forms a complex pathogenic network that drives progressive dopaminergic neuronal loss [61].

## Magneto-proteins: definition and mechanisms

Magneto-proteins are a class of naturally occurring or engineered proteins that respond to magnetic fields, enabling the remote modulation of cellular and neuronal processes. By combining magnetic sensitivity with biologically functional proteins, these constructs allow non-invasive and targeted control of intracellular signalling, gene expression, or ion channel activity. The main types of magneto-proteins include magnetoreceptors, magnetite-forming proteins, ferritin-based magneto-proteins, magnetosensory complex proteins, magnetogenetic actuators, and iron-sulphur cluster proteins [62].

The mechanisms underlying magneto-protein function include magnetothermal effects, in which iron-rich domains absorb energy from alternating magnetic fields to generate localized heating; mechanical torque, where magnetic domains exert forces on protein structures or ion channels; radical pair mechanisms, as observed in CRYs, where magnetic fields modulate spin-dependent biochemical reactions; and ion channel coupling, in which engineered fusion proteins link magnetic sensitivity directly to ionic fluxes and neuronal excitability [7].

Several notable magneto-proteins have been developed and characterized in recent years. Magneto, a synthetic fusion of the mechanosensitive ion channel TRPV4 and ferritin, enables magnetic-field-induced neuronal activation via calcium influx. The MagR complex, a naturally occurring magnetoreception assembly composed of the iron-sulphur cluster protein MagR and Cry4, is implicated in biological magnetosensing and has been adapted for bioengineering applications. Additionally, engineered ferritin variants with enhanced magnetic properties have been designed as versatile platforms for neuromodulation and targeted therapeutic delivery [7,63,64].

By harnessing their unique magnetic field-responsive properties, magneto-proteins offer a novel and versatile toolkit at the interface of molecular engineering, neuroscience, and nanomedicine, with significant potential for translational applications in neurodegenerative disorders such as AD and PD.

### Magnetoreceptors

Magnetoreceptors are proteins or protein complexes that enable cells or organisms to detect and respond to magnetic fields. They function as biological sensors, converting magnetic field information into biochemical or electrical signals. The mechanisms underlying magnetic sensation in living organisms have been extensively studied. Magnetotactic bacteria, for example, synthesize single-domain magnetite crystals approximately 30 nm in size, each containing about one million iron atoms, and organize them into protein-based chains within the cell. The iron atoms interact strongly and align coherently in the same direction, forming a permanent magnetic dipole that functions as a miniature compass at room temperature. The long axis of the cell aligns with the external magnetic field, allowing the bacteria to navigate along magnetic field lines using their flagella [65,66].

Magnetic sensitivity in animals similarly relies on a compass-like mechanism. For instance, the attachment of a small magnetic crystal to a membrane ion channel can confer magnetic sensation. Alternatively, magnetic fields may act directly on specific biochemical reactions involving individual biomolecules. In this process, known as the “radical pair” mechanism, electron-transfer reactions generate radical pairs that can interconvert between singlet and triplet states. Magnetic fields can influence these electron transitions, thereby enabling organisms to detect magnetic fields [67,68].

In other organisms, a class of protein macromolecules acts as magnetoreceptors, first identified in the fruit fly and known as CRYs. These proteins form rod-shaped complexes containing approximately 40 iron atoms distributed along a length of 24 nanometers. Each complex possesses an intrinsic magnetic moment, large enough to align with the geomagnetic field (GMF). Although there is considerable interest in developing single-molecule magnets, their magnetic moments become unstable at temperatures above 14 K due to thermal fluctuations. Consequently, the amount of iron present in CRY proteins appears far below that required for stable magnetic behaviour at physiological temperatures [69].

For a long time, how living organisms sense and respond to the Earth’s magnetic field remained a subject of debate, until studies elucidated the mechanisms underlying the magnetic sensitivity of CRYs and the detection of GMFs in migratory birds and many other species. CRYs are intracellular flavoproteins that respond to UV-A or blue light. Two genes, CRY1 and CRY2, encode the corresponding CRY proteins, CRY1 and CRY2. CRYs are evolutionarily ancient and highly conserved, found across a wide range of organisms, from flies to birds and humans [70].

The magnetoreceptor complex formed by CRY flavoproteins consists of two functional modules: a light-sensing module and a magnetic-sensing module. The light-sensing module contains CRY proteins with conserved photolyase-related domains bound to the cofactor flavin adenine dinucleotide (FAD), located on the outer portion of the complex. The magnetic-sensing module is composed of MagR proteins, which contain iron-sulphur (Fe-S) centres positioned in the inner portion of the complex [63,71].

When exposed to blue or UV-A light, the FAD cofactor in CRYs transitions from its ground state to an excited state and is subsequently converted into flavosemiquinone forms (FAD•⁻ and FADH•). Electron transfer from the flavosemiquinone to adjacent conserved tryptophan residues generates long-lived tryptophan radical pairs. These electrons are then transferred from the tryptophan radicals to the Fe-S centres, ultimately forming a protein-based radical pair capable of exhibiting highly specific responses to magnetic fields [72,73].

Due to their pronounced sensitivity to the weak GMF, CRYs are regarded as biological compasses. These flavoproteins have been identified as key components in orientation, navigation, migration, and even reproduction in many birds, insects, and certain marine animals, such as whales [71].

CRYs mediate cellular responses to environmental stress by activating intracellular signalling pathways, thereby influencing key physiological, metabolic, and growth-related processes. They are expressed in all human cells and participate in indirect DNA repair pathways, contributing to the repair of UV-induced damage as well as the recognition and repair of DNA strand breaks. Notably, CRY expression levels are significantly higher in cancer cells compared to normal cells, and they are considered risk factors in various cancers, including breast, skin, lung, ovarian, prostate, and colorectal cancers [74-76].

CRYs possess the molecular features necessary to function as magnetic sensors. Genetic studies in invertebrates have confirmed the role of CRY in magnetosensation. Several behavioural studies in vertebrates have shown that extremely low-frequency electromagnetic fields, which influence electron spin states, can disrupt magnetic orientation. For example, Fedele et al. demonstrated that exposure to low-frequency electromagnetic fields (300 μT, 50 Hz) shortens the circadian period and induces hyperactivity in fruit flies-effects that disappear in the absence of CRY. Moreover, CRY is expressed in all major organs of birds and plays a role in regulating circadian rhythms in vertebrates.

Additional experiments have shown that fruit flies lacking CRY protein exhibit no magnetic orientation, further supporting the role of this protein in animal magnetoreception. Although CRY responds to magnetic fields via the radical-pair mechanism, the protein alone is theoretically insufficient to form a complete biological compass. Therefore, it is likely that an additional component works alongside CRY to enable organisms to detect and interpret the surrounding magnetic field [77].

Based on laboratory studies and in-silico analyses, the magnetic receptor within CRY-based magnetoreceptor complexes-referred to as MagR-is a rod-shaped protein around which the CRY protein is wrapped. This protein is structurally and magnetically unique, making it a particularly intriguing component of the magnetoreceptor complex. Although it is widely assumed that magnetic receptors are in the retinas of animals, there is no conclusive evidence that any specific cell type, such as cone photoreceptors, is directly sensitive to magnetic stimuli. In fact, both magnetite-based receptors and radical-pair-based receptors possess strong theoretical and experimental foundations, even though neither mechanism appears to be exclusive. Nevertheless, the evidence available across different species may support one hypothesis over the other depending on the organism [63].

### Magnetic structure of cryptochromes

CRYs require linear polymerization with MagR proteins to sense the relatively weak GMF and function as a biological compass capable of detecting magnetic cues. The large hydrodynamic radii observed for both purified MagR proteins and CRY/MagR complexes in chromatographic analyses indicate the occurrence of polymerization. This polymerization, or the intrinsic self-assembly capacity of MagR, represents a critical feature of the magnetic-field sensing system. Such assembly may serve as an amplification mechanism in biological systems, enabling the detection of extremely weak magnetic fields, including the GMF [63].

The link between light sensing and magnetic reception is established through the interaction between CRY and MagR, whereby light-activated CRY is required to generate or modulate the biological compass. A study by Marley and colleagues demonstrated that inhibition of MagR gene expression disrupts circadian behaviour in fruit flies. Similar to observations reported for CRY, the effects of MagR on circadian resetting and light sensitivity suggest a functional connection between magnetoreception, light responsiveness, and circadian regulation. Furthermore, evidence indicates that this magnetoreceptor complex is localized within the cytoplasm, as none of its constituent proteins have been found to be membrane-associated [78].

The fully assembled CRY/MagR complex adopts a rod-like architecture in which the magnetic receptor MagR is positioned at the core, enabling the sensing of surrounding magnetic fields. The light-sensitive, rod-shaped CRY molecules are arranged around this central scaffold, functioning analogously to an antenna that receives optical stimuli. Experimental studies have shown that deletion of the conserved C-terminal helix of CRY markedly reduces the formation of the CRY/MagR complex. Similarly, removal of the iron-sulphur (Fe-S) clusters in MagR almost completely abolishes its interaction with CRY, indicating that these clusters are essential for proper assembly of the complex.

Within the 20-24 nm rod-like model of the CRY magnetoreceptor complex, 20 Fe-S clusters derived from 20 MagR monomers occupy the central region. Every four MagR subunits assemble into a disk-like unit containing four Fe-S clusters arranged in a ring, referred to as an “iron ring”-oriented perpendicular to the long axis of the magnetoreceptor. Two conserved helices of MagR are exposed on the polymer surface and adopt a ladder-like arrangement, serving as the primary interface for CRY binding through helix-helix interactions. The complete CRY/MagR complex comprises 20 MagR cores and 10 CRY molecules, indicating that polymer assembly is highly ordered and tightly regulated [63,67].

### Radical pair model in cryptochromes

According to the radical pair model proposed by Ritz et al., magnetic field detection in living organisms is mediated by photochemically generated radical pairs, in which the flavin adenine dinucleotide (FAD) cofactor within CRY contains an electron with an unpaired spin. Upon photon excitation, these radical pairs are initially formed in a singlet state and can interconvert between singlet and triplet states under the influence of external magnetic fields. This singlet-triplet interconversion alters downstream chemical reaction pathways, thereby enabling organisms to extract directional information from the GMF [79].

The radical pairs generated through this mechanism are inherently unstable and may give rise to distinct chemical products, which ultimately influence navigational behaviour. Importantly, numerical and theoretical analyses have demonstrated that fluctuations in the GMF can modulate the efficiency and persistence of the singlet state, with the lifetime of this state being dependent on the orientation of the magnetic field. In addition, nuclear spins can generate local magnetic fields through hyperfine interactions and may therefore act as internal magnetic references. The magnitude of nuclear spin effects and dipole-dipole coupling between nuclear spins and the surrounding electron cloud are considered critical determinants of magnetic sensitivity in biological systems [67,79,80].

Although the radical pair model provides a compelling quantum-biological framework for magnetoreception, CRY alone is unlikely to generate a sufficiently robust signal to account for reliable magnetic sensing under physiological conditions. The extremely weak strength of the GMF, combined with thermal noise at body temperature and the transient lifetime of radical pairs, presents a significant challenge for direct neural encoding. From a neuroscience perspective, this limitation is particularly important, as magnetic information must ultimately be translated into changes in neuronal excitability, synaptic transmission, or network-level activity [81].

Consequently, additional molecular or structural components are thought to be required to amplify or stabilize magnetic signals before they can influence neuronal processes. Emerging experimental and computational evidence suggests that MagR may serve as such an amplification element by organizing CRY molecules into ordered polymeric assemblies and providing a magnetically responsive scaffold enriched with iron-sulphur (Fe-S) clusters. This cooperative structural arrangement may prolong radical pair lifetimes, enhance spin coherence, or generate localized magnetic fields capable of modulating CRY signalling [63].

Through this integrated mechanism, light-dependent radical pair chemistry is effectively coupled to MagR-based magnetic amplification, enabling weak environmental magnetic cues to be converted into biologically meaningful signals. This coupling provides a plausible pathway by which magnetic information can influence circadian regulation, orientation behaviour, and higher-order neural circuit function, thereby linking quantum-level spin dynamics to systems-level neuroscience phenomena [82,83].

### Role of cryptochromes in regulating circadian rhythms

Circadian rhythms are intrinsic, endogenous cycles present in nearly all eukaryotic organisms. Through the regulation of biological clocks, these rhythms govern daily biochemical, physiological, and behavioural processes and are tightly synchronized with external geophysical cues, particularly the 24-hour light-dark cycle. Disruption of circadian regulation has been shown to adversely affect organismal fitness, and in animals, impaired circadian control is directly associated with reduced survival [84].

At the molecular level, CRYs function as core components of the transcription-translation feedback loop that underlies circadian oscillations. In mammals, CRY1 and CRY2 act as transcriptional repressors by interacting with PERIOD (PER) proteins and inhibiting CLOCK/BMAL1-mediated gene expression, thereby maintaining circadian periodicity and robustness of rhythmic gene expression (83). Genetic studies have demonstrated that CRY1 and CRY2 exert distinct regulatory effects on circadian timing: deletion of Cry1 or Cry2 differentially alters intrinsic circadian period length, whereas simultaneous loss of both proteins results in complete arrhythmicity [85,86].

Experimental evidence further suggests that the light-dependent oxidation of CRYs, leading to the formation of reduced flavin states, represents a potential initiating step in radical pair generation. This mechanism has been implicated in magnetic sensitivity and orientation behaviours in birds and fruit flies. In contrast, mammalian CRYs regulate circadian rhythms largely through light-independent pathways, underscoring fundamental species-specific differences in CRY signalling and functional integration with sensory inputs [87].

Beyond transcriptional regulation, CRYs also participate in post-transcriptional and post-translational control mechanisms that fine-tune circadian oscillations. Regulation of mRNA stability, protein turnover, and subcellular localization of CRY proteins contributes to the precision and adaptability of circadian rhythms across different tissues and developmental stages. Notably, developmental studies indicate that rhythmic CRY1 expression during early postnatal periods plays a critical role in establishing stable circadian period length later in life, highlighting an important link between circadian timing and neurodevelopment [88,89].

In mammals, CRYs exert their central regulatory functions in the suprachiasmatic nucleus (SCN), the brain's master circadian pacemaker. Within SCN neurons, CRYs influence electrophysiological depolarization, resting membrane potential, action potential firing, and neuronal responsiveness to photic input. Emerging evidence further suggests that CRYs may modulate neural sensitivity to GMF-related cues, providing a potential interface between circadian regulation, environmental magnetic signals, and neuronal excitability [90,91].

As essential components of the molecular circadian clock, CRYs regulate a broad range of biological processes beyond daily timekeeping, including cell cycle progression, DNA repair and replication, transcriptional control, and cellular growth and differentiation. Importantly, accumulating epidemiological and experimental data indicate that circadian disruption is associated with adverse health outcomes, such as sleep and mood disorders, metabolic syndrome, gastrointestinal diseases, and increased susceptibility to tumour initiation and progression. Dysregulation of CRY expression and function has been implicated in multiple cancer types, emphasizing their relevance to both neural homeostasis and disease pathogenesis [92,93].

## Magnetoreceptors in Alzheimer’s disease

AD is a progressive neurodegenerative disorder characterized by synaptic dysfunction, cognitive decline, and selective neuronal vulnerability in the hippocampus and cortex. While extracellular Aβ plaques and intracellular NFTs composed of hyperphosphorylated tau define AD pathology, accumulating evidence highlights mitochondrial dysfunction, oxidative stress, impaired proteostasis, neuroinflammation, and circadian dysregulation as convergent drivers of disease progression. In this context, emerging studies suggest that magnetoreceptors, particularly CRY/MagR complexes, may influence neuronal redox homeostasis, mitochondrial metabolism, synaptic function, and circadian timing, providing a potential mechanistic interface between magnetic field sensitivity and neurodegenerative processes [94-96].

CRY/MagR complexes operate through radical-pair chemistry, generating spin-correlated electrons within FAD and adjacent aromatic residues. Perturbations in radical-pair dynamics can modulate intracellular redox signalling and ROS production. In metabolically active cortical and hippocampal neurons, even modest elevations in ROS can exacerbate mitochondrial electron leakage, disrupt cardiolipin integrity and impair ATP synthesis, amplifying oxidative stress and calcium dysregulation. Redox-sensitive calcium channels, including voltage-gated calcium channels and the mitochondrial calcium uniporter, may further couple oxidative stress to intracellular calcium overload, promoting activation of proteases and kinases implicated in AD pathology [97-99].

Oxidative modifications of Aβ, including methionine oxidation and tyrosine cross-linking, enhance β-sheet formation and oligomer stability, while redox-dependent modulation of β- and γ-secretase activity can bias APP processing toward the amyloidogenic pathway. Similarly, tau phosphorylation is highly sensitive to oxidative stress, as ROS-activated kinases including GSK3β, CDK5, and stress-activated MAPKs promote tau hyperphosphorylation, whereas oxidative inhibition of phosphatases such as PP2A prolongs pathological tau states. These mechanisms favour tau misfolding, paired helical filament formation, and NFTs accumulation [100-102].

Beyond protein aggregation, radical-pair-induced redox perturbations can compromise neuronal proteostasis. Oxidative damage to molecular chaperones, autophagy regulators, and lysosomal enzymes impairs the clearance of misfolded Aβ and tau, reinforcing a feed-forward loop of mitochondrial dysfunction and proteostasis failure. At the network level, ROS-sensitive ion channels and redox-modulated signalling pathways can alter neuronal excitability, synaptic plasticity, and long-term potentiation, providing a mechanistic link between magnetic field-dependent radical-pair signalling and synaptic dysfunction in AD [103-105].

CRYs are also core components of the molecular circadian clock. CRY-dependent circadian regulation orchestrates neuronal metabolism, mitochondrial dynamics, antioxidant defences, and synaptic homeostasis in a time-of-day-dependent manner. Disruption of CRY oscillations can impair sleep-wake cycles, reduce slow-wave sleep, and desynchronize neuronal and glial populations. Circadian dysfunction has been strongly associated with impaired glymphatic clearance of metabolic waste, including Aβ, primarily during sleep. Altered circadian control of kinase and phosphatase activity may bias tau toward hyperphosphorylation, while circadian disruption of autophagy and lysosomal pathways further impairs proteostasis. These observations suggest that circadian misalignment and radical-pair-dependent redox perturbations are interconnected mechanisms that converge on neuronal vulnerability in AD [95,106].

Importantly, emerging evidence indicates that external magnetic fields can modulate CRY activity, providing a potential means to influence CRY/MagR signalling in neural tissue. In Drosophila, exposure to moderate static magnetic fields (~100 mT) potentiates blue-light-activated CRY signalling, resulting in enhanced neuronal depolarization and increased action potential firing in a CRY-dependent manner. Additionally, field strengths slightly above the ambient GMF (*e.g.* 500 μT) have been shown to influence the phosphorylation state and conformational activity of CRY1 and CRY2 proteins, modifying their photoreceptive output. Behavioural studies indicate that alternating or static magnetic fields can also modulate memory formation, learning, and neural excitability in a CRY-dependent fashion, highlighting a mechanistic link between magnetoreception and neural circuit function [78,90,107].

While direct causal evidence in mammalian neurons or human AD models remains limited, these findings suggest that external magnetic fields could modulate CRY-mediated radical-pair signalling, redox homeostasis, mitochondrial metabolism, synaptic plasticity, and circadian regulation processes central to AD pathology. Conceptually, magnetic field modulation of CRY/MagR activity could influence oxidative stress, Aβ clearance, tau phosphorylation, and synaptic function, providing a non-invasive approach to probe or potentially alter neurodegenerative pathways. Further studies in mammalian and human-relevant models are required to determine whether these effects are physiologically significant and therapeutically actionable [108,109].

## Magnetoreceptors in Parkinson’s disease

PD is a progressive neurodegenerative disorder primarily affecting dopaminergic neurons in the substantia SNpc, resulting in characteristic motor symptoms and a wide spectrum of non-motor manifestations. Core pathological features of PD include mitochondrial dysfunction, oxidative stress, impaired proteostasis, and aggregation of α-synuclein into LBs. As outlined in earlier sections, magnetoreceptors-particularly the CRY/MagR complex-operate through radical-pair-mediated redox mechanisms and circadian regulation. In the context of PD, these pathways intersect with molecular processes that are especially critical for dopaminergic neuron survival and function [110,111].

Through their impact on spin-dependent electron transfer, CRY/MagR assemblies may indirectly shape cellular redox homeostasis. Neuronal populations with elevated energetic demands, such as dopaminergic neurons, are particularly sensitive to redox imbalance because of sustained pacemaking activity and tight coupling between mitochondrial function and calcium fluxes. Under these conditions, minor disruptions in radical-pair processes could trigger excessive ROS accumulation, thereby accelerating oxidative damage and increasing susceptibility to neurodegeneration in PD [112].

Mitochondrial dysfunction represents a central pathogenic axis in PD, particularly involving impairment of complex I activity in SNpc neurons. Redox perturbations associated with magnetoreceptor signalling may further destabilize mitochondrial electron transport, increasing electron leakage and ROS generation. Elevated ROS promotes oxidative damage to mitochondrial DNA, cardiolipin peroxidation, and depolarization of the mitochondrial membrane potential, ultimately compromising ATP synthesis and calcium buffering. These effects are especially deleterious in dopaminergic neurons, where sustained calcium-dependent firing imposes continuous bioenergetic stress [113,114].

α-synuclein aggregation is a defining pathological hallmark of PD. Oxidative modifications of α-synuclein, including methionine oxidation, tyrosine nitration, and redox-dependent conformational destabilization, enhance its propensity to misfold and form toxic oligomeric assemblies. Radical-pair-associated increases in ROS may therefore indirectly accelerate α-synuclein oligomerization and fibrillization. In addition, oxidatively modified α-synuclein exhibits increased affinity for mitochondrial membranes, where it can inhibit complex-I function and further exacerbate mitochondrial dysfunction, establishing a self-reinforcing pathogenic loop [115].

Redox-sensitive kinase and phosphatase signalling pathways provide another point of convergence between magnetoreceptor activity and PD pathology. Oxidative stress activates kinases such as GSK3β, JNK, p38 MAPK, and LRRK2, which are implicated in pathological α-synuclein phosphorylation, particularly at Ser129 a modification strongly associated with LBs formation. Concurrently, ROS-mediated inhibition of phosphatases such as PP2A prolongs aberrant phosphorylation states. Together, these processes favor sustained α-synuclein aggregation and disrupt cytoskeletal integrity and axonal transport in dopaminergic neurons [116,117].

Failure of protein quality control mechanisms further accelerates neurodegeneration in PD. Both the ubiquitin-proteasome system and the autophagy-lysosomal pathway are essential for α-synuclein clearance yet are highly sensitive to oxidative damage. Redox imbalance associated with radical-pair signalling may impair chaperone activity, lysosomal enzyme function, and mitophagy, leading to intracellular accumulation of misfolded α-synuclein. This proteostatic failure amplifies mitochondrial stress and oxidative burden, reinforcing neurodegenerative cascades [118].

Circadian dysregulation is increasingly recognized as an important contributor to PD pathophysiology. Clinical and experimental studies indicate that disturbances in circadian rhythms manifesting as fragmented sleep-wake cycles, altered melatonin secretion, and impaired diurnal regulation of dopamine synthesis and release often precede or accompany motor symptom onset. At the cellular level, circadian clock components, including CRY1 and CRY2, regulate mitochondrial bioenergetics, antioxidant gene expression, and autophagy in dopaminergic neurons. Disruption of CRY-dependent circadian signalling can therefore exacerbate oxidative stress, impair mitochondrial quality control, and reduce the efficiency of α-synuclein clearance. In the SNpc, circadian misalignment may further increase neuronal vulnerability by uncoupling energy production from intrinsic pacemaking activity [111,119].

Although direct causal evidence linking magnetoreceptor activity to PD in humans remains limited, experimental studies in model organisms demonstrate that external magnetic fields can modulate CRY-dependent redox signalling, neuronal excitability, and circadian behaviour. Collectively, these findings suggest that CRY/MagR-mediated mechanisms may intersect with mitochondrial dysfunction, oxidative stress, α-synuclein aggregation, impaired proteostasis, and circadian disruption-core processes underlying PD neurodegeneration. Further studies in mammalian and human-relevant models are required to determine whether modulation of magnetoreceptor pathways represents a meaningful contributor to disease progression or a potential target for non-invasive neuromodulatory strategies [90,120]. A summary of the key physiological roles discussed in the context of AD and PD is provided in Table 1, highlighting their potential relevance to CRY/MagR-mediated pathways in neurodegeneration.

**Table 1.** Key proteins, their physiological and pathological roles, and potential interactions with CRY/MagR magnetoreceptors in Alzheimer’s and Parkinson’s diseases

Disease	Protein / Molecule	Physiological role	Pathological role	Interaction with CRY/MagR / magnetic fields
AD	Amyloid-β (Aβ)	Synaptic plasticity, memory, antimicrobial activity	Plaque formation, synaptic toxicity, calcium and mitochondrial dysregulation	Radical-pair-mediated ROS may enhance Aβ aggregation and tau phosphorylation
AD	Tau	Microtubule stabilization, axonal transport	Neurofibrillary tangles (NFTs), hyperphosphorylation, mitochondrial and synaptic dysfunction	Redox stress via CRY/MagR can promote tau hyperphosphorylation and aggregation
PD	α-Synuclein	Synaptic vesicle regulation, dopamine release	Lewy body formation, synaptic dysfunction, oxidative stress	ROS from radical-pair signaling may accelerate α-synuclein aggregation
PD	Dopaminergic neurons	Dopamine synthesis, pacemaking activity	Neuronal death, metabolic and oxidative stress	Highly sensitive to CRY/MagR-mediated redox perturbations, increasing vulnerability
AD & PD	Mitochondria	ATP production, calcium buffering	Dysfunction, increased ROS	CRY/MagR radical-pair activity can modulate ROS levels and mitochondrial function
AD & PD	Circadian clock (CRY1/CRY2)	Regulation of circadian rhythms, gene expression, cellular metabolism	Circadian disruption, impaired protein clearance	Directly influenced by magnetic fields through CRY/MagR, affecting neuronal metabolism and proteostasis

## Conclusions

AD and PD exemplify the urgent translational challenge of neurodegeneration: despite decades of mechanistic insight, therapeutic progress has largely stalled at symptomatic management. The convergence of protein aggregation, mitochondrial failure, oxidative stress, disrupted proteostasis, and circadian misalignment underscores the need for fundamentally new strategies that move beyond conventional pharmacology and invasive neuromodulation.

Magneto-proteins introduce a conceptual shift in how neuronal processes may be interrogated and controlled. By endowing biologically active proteins with magnetic field sensitivity, these systems enable remote, non-invasive, and spatiotemporally precise modulation of intracellular signalling networks. Among them, CRY-based magnetoreceptors-and particularly the CRY/MagR complex-stand out as uniquely positioned at the intersection of magnetic sensing, redox biology, and circadian regulation. This multifunctional integration directly overlaps with molecular pathways that define vulnerability in both AD and PD.

Evidence from experimental models indicates that CRY/MagR-mediated mechanisms can influence mitochondrial bioenergetics, ROS production, protein aggregation dynamics, autophagic flux, and circadian control of neuronal metabolism. In dopaminergic and cortical neurons, where energetic demand and oxidative burden are intrinsically high, even modest modulation of these pathways may exert outsized effects on disease trajectories. Although definitive validation in human systems is still lacking, the coherence between magnetoreceptor biology and neurodegenerative pathophysiology is difficult to ignore.

Moving forward, the field must prioritize rigorous mechanistic validation in mammalian models, quantitative assessment of magnetic field-protein interactions, and integration with gene delivery and precision neuromodulation technologies. If successfully translated, magneto-proteins could redefine non-invasive neuromodulation, not merely as a tool for symptom control, but as a platform for targeting core disease mechanisms in neurodegenerative disorders. Such a paradigm shift has the potential to reshape both our understanding and treatment of disorders that remain among the most intractable challenges in modern neuroscience.

## Abbreviations

Aβ: Amyloid-β
ABCB1: ATP-binding cassette transporter P-glycoprotein
AD: Alzheimer’s disease
AICD: APP intracellular domain
APPL: Amyloid precursor protein-like
APLP1: Amyloid precursor-like protein 1
APLP2: Amyloid precursor-like protein 2
APP: Amyloid precursor protein
BMAL1: Brain and muscle ARNT-like protein 1
C83: 83-amino-acid C-terminal fragment of APP
C99: 99-amino-acid C-terminal fragment of APP
CDK5: Cyclin-dependent kinase 5
CK1: Casein kinase 1
CK2: Casein kinase 2
CRY: Cryptochrome
CRY1: Cryptochrome 1
CRY2: Cryptochrome 2
FAD: Flavin adenine dinucleotide
Fe-S: Iron-sulfur
GBA1: Glucocerebrosidase gene
GSK3: Glycogen synthase kinase-3
GSK3β: Glycogen synthase kinase-3 beta
GMF: Geomagnetic field
IDE: Insulin-degrading enzyme
JNK: c-Jun N-terminal kinase
LB: Lewy body
LRP1: Low-density lipoprotein receptor-related protein 1
LRRK2: Leucine-rich repeat kinase 2
LAMP2A: Lysosome-associated membrane protein 2A
MagR: Magnetic receptor protein
MAPK: Mitogen-activated protein kinase
mT: Millitesla
μT: Microtesla
NAC: Non-amyloid-β component
NDs: Neurodegenerative diseases
NEP: Neprilysin
NFT: Neurofibrillary tangle
PD: Parkinson’s disease
PER: Period protein
PINK1: PTEN-induced kinase 1
PLK2: Polo-like kinase 2
PP2A: Protein phosphatase 2A
PP2B: Protein phosphatase 2B
PTM: Post-translational modification
RAGE: Receptor for advanced glycation end products
REM: Rapid eye movement
ROS: Reactive oxygen species
SCN: Suprachiasmatic nucleus
SNARE: Soluble N-ethylmaleimide-sensitive factor attachment protein receptor
SNCA: Synuclein alpha gene
SNpc: Substantia nigra pars compacta
TRPV4: Transient receptor potential vanilloid 4
